# Results of simultaneous radon and thoron measurements in 33 metropolitan areas of Canada

**DOI:** 10.1093/rpd/ncu141

**Published:** 2014-04-19

**Authors:** Jing Chen, Lauren Bergman, Renato Falcomer, Jeff Whyte

**Affiliations:** Health Canada, Radiation Protection Bureau, 775 Brookfield Road, Ottawa, Canada, K1A 1C1

## Abstract

Radon has been identified as the second leading cause of lung cancer after tobacco smoking. ^222^Rn (radon gas) and ^220^Rn (thoron gas) are the most common isotopes of radon. In order to assess thoron contribution to indoor radon and thoron exposure, a survey of residential radon and thoron concentrations was initiated in 2012 with ∼4000 homes in the 33 census metropolitan areas of Canada. The survey confirmed that indoor radon and thoron concentrations are not correlated and that thoron concentrations cannot be predicted from widely available radon information. The results showed that thoron contribution to the radiation dose varied from 0.5 to 6 % geographically. The study indicated that, on average, thoron contributes ∼3 % of the radiation dose due to indoor radon and thoron exposure in Canada. Even though the estimated average thoron concentration of 9 Bq m^−3^ (population weighted) in Canada is low, the average radon concentration of 96 Bq m^−3^ (population weighted) is more than double the worldwide average indoor radon concentration. It is clear that continued efforts are needed to further reduce the exposure and effectively reduce the number of lung cancers caused by radon.

## INTRODUCTION

Radon is a naturally occurring radioactive gas generated by the decay of uranium- and thorium-bearing minerals in rocks and soils. Radon and its decay products are the major contributors to human exposure from natural radiation sources^(1)^. Radon has been identified as the second leading cause of lung cancer after tobacco smoking^(2)^. ^222^Rn (radon gas) and ^220^Rn (thoron gas) are the most common isotopes of radon.

**Figure 1.**
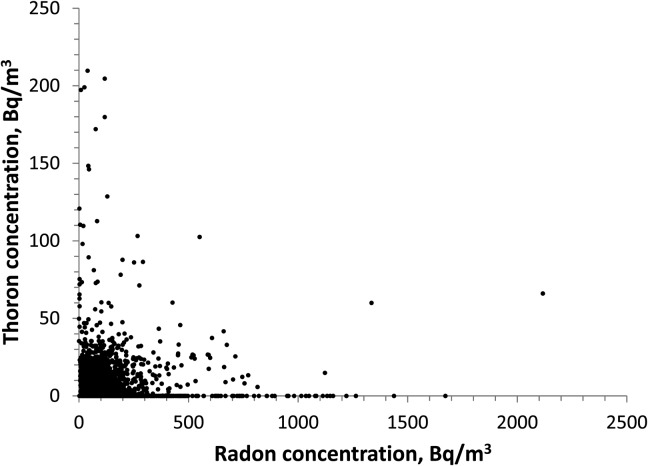
Result distribution of paired radon–thoron measurements in 3215 Canadian homes.

Radon is a member of the ^238^U decay chain whereas thoron is a member of the ^232^Th decay chain. Because radon and thoron are members of different decay chains, their concentrations depend in part on the uranium and thorium levels in local soils and building materials. Because wood-frame construction is very popular for family houses, building materials contribute very little to indoor radon and thoron in Canada. Information on geographical distributions of uranium and thorium in the ground is limited or not available for many metropolitan areas of Canada, as shown in Figures 1 and 2 of a previous publication^(3)^. Nevertheless, the previous study was able to demonstrate that average indoor radon and thoron concentrations in an area correlate well with the average uranium and thorium concentrations in the ground, even though the ratio of uranium and thorium concentrations could vary significantly from one area to another.

Many studies have confirmed that there is no clear correlation between radon and thoron concentrations and that thoron concentrations could not be predicted from widely available radon information^(4–10)^. In 2008 and 2011, simultaneous radon and thoron measurements were performed in a total of 370 Canadian homes in five communities^(5–7)^. Based on those results, it was estimated that thoron contributes ∼8 % of the radiation dose due to indoor radon and thoron exposure^(3)^. To confirm this estimate, a larger cross-Canada survey was needed.

The Cross-Canada Survey of Radon Concentrations in Homes study was completed recently^(11)^. However, detectors used in that study were not sensitive to thoron, and thoron information was not included as part of the radon survey^(12)^.

In 2012, another survey was initiated in Canadian metropolitan areas to estimate thoron contribution to indoor radon and thoron exposure^(13)^. Results of this 2012/2013 radon–thoron survey are reported here.

## METHODS

In order to assess thoron contribution to indoor radon and thoron exposure combined, a survey of residential radon and thoron concentrations was designed for ∼4000 homes in all 33 census metropolitan areas (CMAs) specified by Statistics Canada^(14)^, which cover ∼70 % of the Canadian population. The CMAs, from east to west, are St. John's, Halifax, Moncton, Saint John, Saguenay, Québec, Sherbrooke, Trois-Rivières, Montréal, Ottawa-Gatineau, Kingston, Peterborough, Oshawa, Toronto, Hamilton, St. Catharines—Niagara, Kitchener–Cambridge–Waterloo, Brantford, Guelph, London, Windsor, Barrie, Greater Sudbury, Thunder Bay, Winnipeg, Regina, Saskatoon, Calgary, Edmonton, Kelowna, Abbotsford-Mission, Vancouver and Victoria. As the Cross-Canada Survey of Radon Concentrations in Homes, there were several qualifying criteria that had to be satisfied for a participant to be eligible to take part in the study. First, participants had to be the head of the household and 18 y of age or older. Participants also had to be homeowners and be living in their primary residence. People who rented a home were not included in the study because there is no requirement on the part of landlords to remediate high radon–thoron levels if they are found in a home. In addition, participants could not live on military bases or on-reserve, since these homes were, or were expected to be, covered in other surveys. Homes that were built on stilts or high-rise condo units that were above the second floor did not qualify. Finally, homeowners could not have planned to move or be away during the proposed timeline of the study.

The survey followed the procedure outlined in Health Canada's guide for radon measurements in residential dwellings^(15)^. Radon levels in a home can vary significantly over time. In fact, it is not uncommon to see radon levels in a home change by a factor of 2 to 3 over a 1-day period, and variations from season to season can be even larger. The highest radon levels are usually observed during winter months. As a result, a long-term measurement period will give a much better indication of the annual average indoor radon concentration. During a long-term measurement, there are no requirements for the occupants to change their life-style once the measurement devices have been put in place. Therefore, Health Canada recommends the placement of at least one long-term detector in a home for a minimum of 3 to 12 months (12 months is optimal). For periods <12 months, the testing period should include a mix of seasons or be in a mid-season to best provide a measurement that reflects the annual average level. The ideal 3-month testing period would be in the typical heating season that runs from October through to April. The least ideal period is during the summer since open window conditions often prevail.

The study was designed to recruit during the summer of 2012 with the testing to occur in the 2012–2013 fall/winter (October to March) periods. Each CMA was targeted to recruit ∼122 participants. Participants were recruited over the telephone by a contracted market research firm, Prairie Research Associates (PRA), which employed random digit dialing with a sampling product of ASDE Survey Sampler. Test kits were then mailed out in October 2012 to those who agreed to participate.

Instruction was given to all participants that the detector should be deployed in the lowest lived-in level of the home where someone spends at least 4 h a day. In order for the result to be indicative of the average annual radon and thoron exposure, all tests were conducted for a period of at least 3 months. Essential information (the start and stop time of the test) had to be included with the returned detector.

To determine the concentrations of radon and thoron, a passive integrated radon–thoron discriminative detector developed at the National Institute of Radiological Sciences in Japan (commercially known as RADUET) was used in this survey. The principle and technical descriptions of RADUET detectors have been given in previous publications^(5, 16)^. A RADUET contains paired detection chambers, a low-diffusion chamber and a high-diffusion chamber. In principle, the track densities in the low-diffusion chamber (TD_L_) and high-diffusion chamber (TD_H_) depend on both radon and thoron concentrations in the air:

(1) $$\text{T}{\text{D}}_{L}=c_{11}(\text{Rn}-b_{1})+c_{12}(\text{Tn}-b_{2})$$

(2) $$\text{T}{\text{D}}_{H}=c_{21}(\text{Rn}-b_{1})+c_{22}(\text{Tn}-b_{2})$$

where *b*_1_ and *b*_2_ are the background noise levels for radon and thoron concentration, respectively, i.e. they appear as radon or thoron concentration readings for blank control detectors. The values *c*_11_, *c*_12_, *c*_21_ and c_22_ are the calibration coefficients. The low-diffusion chamber limits diffusion of thoron into the chamber, therefore, *c*_11_ >> *c*_12_. The high-diffusion chamber is designed such that both radon and thoron can diffuse into the chamber easily, and *c*_21_ ≈ *c*_22_.

To determine the above-mentioned coefficients, several groups of RADUETs were randomly taken from 5000 detectors (purchased at different times) and exposed to three different known radon and thoron concentrations at the National Institute of Radiological Sciences and Hirosaki University in Japan. The coefficients in Equations 1 and 2 were then determined to be *c*_11_ = 0.029, *c*_12_ = 0.001, *c*_21_ = 0.028, *c*_22_ = 0.022, *b*_1_ = 0.8 and *b*_2_ = 0.7. This calibration is necessary to account for possible changes in the detectors after the storage period and variations in the reader system and/or etching process.

RADUET detectors returned for analysis were etched according to manufacturer's instructions. A commercial alpha-track reader (RadoSys) was used to obtain total track densities in paired low- and high-diffusion chambers (TD_L_ and TD_H_). With these raw data, TD_L_ and TD_H_, and the calibration coefficients determined earlier, radon and thoron concentrations are calculated as the solution of Equations 1 and 2.

Based on the fact that concentration in one chamber depends on the other and the calculation procedure given by Currie^(17)^, the detection limits were estimated to be 3 Bq m^−3^ for radon and 4 Bq m^−3^ for thoron^(18)^. Even though the theoretically estimated detection limits are low, it was decided to only report radon and thoron concentrations of >15 Bq m^−3^ to the survey participants due to uncertainties of many environmental factors and variations in the field deployment. All results below 15 Bq m^−3^ were reported as <15 Bq m^−3^.

As assessed in the previous publication^(3)^, the annual effective dose due to indoor radon exposure for a population in a given area, *E*_Rn_(nSv), was assessed based on the formula given by the UNSCEAR report^(1)^:

(3) $$E_{\mathrm{Rn}}=C_{\mathrm{Rn}}\times 0.4\times 7000\times 9$$

where *C*_Rn_ is the arithmetic mean (AM) radon concentration (in Bq m^−3^), the typical value of 0.4 was used as the equilibrium factor for radon indoors, a recommended value of 9 nSv (Bq m^−3^ h)^−1^ was used to convert radon equilibrium-equivalent concentration (EEC) to population effective dose and an 80 % home occupancy time, i.e. 7000 h, was assumed. The population dose due to indoor radon exposure is proportional to the AM radon concentration in an area.

The population effective dose due to indoor thoron exposure, *E*_Tn_(nSv), was assessed based on the formula given in the UNSCEAR report^(1)^:

(4) $$E_{\mathrm{Tn}}=C_{\mathrm{Tn}}\times 0.02\times 7000\times 40$$

where *C*_Tn_ is the AM thoron concentration (in Bq m^−3^), the typical value of 0.02 was used as the equilibrium factor for thoron indoors and a recommended value of 40 nSv (Bq m^−3^ h)^−1^ was used to convert thoron EEC to the effective dose. As in the case of radon, the population dose due to indoor thoron exposure is proportional to the AM thoron concentration in an area.

The total effective dose is the sum of the effective doses due to exposures to indoor radon and thoron. Thoron contribution to the radiation dose due to indoor radon and thoron exposure can then be determined as follows:

(5) $$\frac{E_{\mathrm{Tn}}}{E_{\mathrm{Rn}}+E_{\mathrm{Tn}}}$$

## RESULTS

During the summer of 2012, the research firm PRA dialled over 100 000 telephone numbers in order to recruit at least 4000 participants from 33 metropolitan areas. About 92.5 % of households phoned were eligible to participate. Overall, the response rate was 13.5 % and the refusal rate was 30.9 %, which were expected for a study of this nature. By the end of October 2012, detectors were mailed to a total of 4064 participants. Detailed breakdown in the numbers of participants for each CMA is given in Table 1. According to the instruction included in the mail-out, the participants deployed the alpha-track radon–thoron detectors in their homes for 3 months, after which they were reminded to mail the detectors back to the Health Canada for analysis. The survey had a return rate of 79 %. Five participants decided to withdraw from the survey. A total of 3215 test results were reported directly to the survey participants. Due to mechanical damage or other technical reasons, test results could not be generated for 11 participants. Among them, nine participants agreed to conduct a re-test during the 2013/2014 test season. For those detectors returned without start and end dates recorded, a test duration of 91 d is assumed. This assumed exposure period was included and explained in the results letter sent to the participants.

**Table 1.** Sample distribution and test results in 33 CMAs.

CMA	Population (thousands)	Number of participants	Results reported	Number of Tn < DL	Rn, Bq m^−3^ AM ± SD	Tn, Bq m^−3^ AM ± SD	*E*_Tn_/ (*E*_Rn_ + *E*_Tn_) (%)
Abbotsford-Mission	178.1	122	90^a^	38	58 ± 53	11 ± 17	4.1
Barrie	196.0	122	88^b^	44	85 ± 88	10 ± 16	2.5
Brantford	140.5	122	89^b^	43	108 ± 96	12 ± 19	2.5
Calgary	1309.2	123	99	64	135 ± 106	6 ± 6	0.9
Edmonton	1230.1	122	97^c^	51	113 ± 68	8 ± 9	1.6
Greater Sudbury	164.0	122	96^a^	56	131 ± 113	8 ± 13	1.4
Guelph	142.9	122	102	59	131 ± 153	8 ± 10	1.3
Halifax	413.7	125	102^b^	48	185 ± 269	12 ± 17	1.4
Hamilton	756.6	122	87	42	85 ± 67	10 ± 20	2.5
Kelowna	184.7	122	108	63	134 ± 135	8 ± 11	1.4
Kingston	165.5	122	97^b^	53	165 ± 131	11 ± 19	1.5
Kitchener–Cambridge–Waterloo	505.1	122	102	55	66 ± 37	9 ± 18	2.8
London	500.0	122	81^b^	43	85 ± 60	7 ± 8	1.9
Moncton	143.0	122	95^c^	47	77 ± 77	8 ± 11	2.3
Montréal	3957.7	122	99^a^	48	120 ± 143	9 ± 9	1.6
Oshawa	375.6	122	96^b^	42	61 ± 48	12 ± 16	4.0
Ottawa-Gatineau	1273.3	122	109	61	108 ± 91	11 ± 21	2.2
Peterborough	122.4	122	100	52	100 ± 60	8 ± 7	1.7
Québec	769.6	122	99^a^	58	115 ± 152	9 ± 22	1.7
Regina	226.3	121	96	69	302 ± 254	7 ± 12	0.5
Saguenay	152.6	124	100	44	89 ± 104	11 ± 21	2.6
Saint John	128.9	154	116	54	115 ± 131	13 ± 24	2.4
Saskatoon	284.0	122	104	65	152 ± 78	9 ± 13	1.3
Sherbrooke	203.5	122	104^b^	60	238 ± 344	8 ± 9	0.8
St. Catharines—Niagara	405.8	122	87^a^	32	56 ± 36	11 ± 12	4.3
St. John's	200.6	123	97^c^	52	88 ± 63	7 ± 7	1.7
Thunder Bay	127.1	122	94	53	156 ± 133	10 ± 16	1.4
Toronto	5941.5	123	91^b^	33	57 ± 37	9 ± 8	3.3
Trois-Rivières	148.3	122	95^b^	30	43 ± 22	11 ± 12	5.4
Vancouver	2463.7	121	98^a^	40	28 ± 25	8 ± 7	6.0
Victoria	363.1	122	102	46	37 ± 26	8 ± 8	4.5
Windsor	333.4	122	94^b^	55	154 ± 121	11 ± 23	1.6
Winnipeg	778.4	122	101	50	257 ± 210	8 ± 9	0.7
Total or average	24 285	4064	3215	48 %	96 ± 87	9 ± 11	2.7

^a^In two cases, only radon results were available.

^b^In one case, only radon result was available.

^c^In three cases, only radon results were available.

Duplicate detectors were placed side-by-side in every ten homes for quality control purposes. A total of 319 duplicates were returned. Twelve duplicates had one of the two RADUETs damaged. Radon concentrations measured with those randomly distributed duplicates ranged from 7 to 1175 Bq m^−3^ with an average of 104 Bq m^−3^ whereas thoron concentrations were much lower and ranged from non-detectable to 164 Bq m^−3^ with an average of only 8 Bq m^−3^. Good agreement in radon results was obtained from the duplicates placed side-by-side. The average relative deviation of radon concentrations was 8 %. However, a rather large deviation of 78 % in thoron concentrations was observed among the same duplicates. This is mainly due to thoron concentrations that were below the detection limit in about half of homes surveyed. In those cases, one detector showed non-detectable whereas the duplicate recorded a value slightly above the detection limit. It should also be mentioned that detectors have finite dimensions and can be >10 cm apart even when placed side-by-side and bound together. Because of its short half-life, the thoron measurement is more sensitive to variations in air flow around detectors, and more dependent on distances from thoron sources. This can explain the relatively large deviation in readings from detectors for thoron concentrations in comparison with radon measurements. For homes with control detectors, average radon and thoron concentrations from those duplicate detectors were reported.

Among the 3215 test-kits returned, both radon and thoron measurements were available for 3184 homes. Thirty-one participants received only radon results due to damage to the high-diffusion chamber. The characteristics of the radon and thoron concentrations in the 33 CMAs are summarised in Table 1. Radon and thoron concentrations in individual homes were not correlated, as shown in Figure 1 for the entire data set. This is also true for individual CMAs.

Radon was present in all homes in varying concentrations with the highest measured concentration of 2117 Bq m^−3^. The population-weighted AM concentration of radon and the standard deviation (SD) were 96 and 87 Bq m^−3^, respectively.

On average (population weighted), 48 % of homes surveyed had thoron concentration below detection limit. As done in previous thoron studies^(5–7)^, a thoron concentration at half of the detection limit, i.e. 2 Bq m^−3^, was assigned to those below the detection limit for the purpose of statistical analysis. Thoron was present in about half of the homes surveyed in this study with the highest measured value being 210 Bq m^−3^. The population-weighted AM concentration of thoron was 9 ± 11 Bq m^−3^.

For each CMA using the measured AM radon and thoron concentrations, thoron contribution to the total indoor radon exposure was determined according to Equation 5. Results are given in Table 1. Thoron contribution to the radiation dose varied widely, ranging from 0.5 to 6.0 % geographically. On average (population weighted), thoron contributes ∼2.7 % of the radiation dose due to indoor radon and thoron exposure in Canada.

## DISCUSSION

Based on limited measurements in <400 homes^(5–7)^, it was estimated that thoron contributes ∼8 % of the radiation dose due to indoor radon and thoron exposure^(3)^. The current study provided simultaneous radon and thoron measurements in >3000 homes in 33 CMAs across Canada. It is confirmed that thoron contributes a small fraction, ∼3 % of the dose from exposure to indoor radon. Consistent with previous thoron studies, thoron was not detectable in about half of the homes surveyed.

Among the 33 metropolitan areas, thoron contributions were determined in three metropolitan areas in previous studies^(5–7)^. They are Halifax, Ottawa-Gatineau (the National Capital Region, NCR) and Winnipeg. Table 2 provides the results of all available radon or radon–thoron studies or surveys in those three areas. Thoron concentrations were significantly lower than the results previously observed in all three cities listed in Table 2. The consistently lower thoron concentrations could be due to different measurement protocols being followed in previous and current studies. In the previous thoron studies^(5–7)^, instructions were given to participants to place RADUET detectors in the basement and within 50 cm of the external foundation wall, in order to increase thoron detection capability. The average distance between detector and foundation wall was 7 cm with an SD of 10 cm, and the average distance between detector and basement floor was 42 cm with an SD of 30 cm in the 2008 Winnipeg thoron study^(6)^.

**Table 2.** Comparison of current results with results from previous studies in Halifax, Ottawa-Gatineau (NCR) and Winnipeg.

	Sample size	Rn > 200 Bq m^−3^ (%)	Radon AM ± SD, Bq m^−3^	Thoron AM ± SD, Bq m^−3^
Halifax				
2011 study	64	32	259 ± 475	50 ± 46
2012 survey	103	14	105 ± 176	—
This study	102	29	185 ± 269	12 ± 17
NCR				
2008 study	95	12	110 ± 168	56 ± 123
2012 survey	126	9.5	88 ± 134	—
This study	109	10	108 ± 91	11 ± 21
Winnipeg				
1990 study				
Basements	3669	31	197 ± 194	—
Bedrooms	4238	13	121 ± 136	—
2009 study	116	20	143 ± 101	34 ± 45
2012 survey	66	12	113 ± 80	—
This study	101	49	257 ± 210	8 ± 9

In the current study, participants were required to follow Health Canada's guide for radon measurements in residential dwellings^(15)^. In addition to other environmental factors, the Guide specifies the preferred detector location being by an interior wall at a height of 0.8–2 m from the floor in the typical breathing zone, however, at least 50 cm from the ceiling and 20 cm from other objects so as to allow normal airflow around the detector. Potential measurement locations include family rooms, living rooms, dens, playrooms and bedrooms. A lower level bedroom is preferred because people generally spend more time in their bedrooms than in any other room in the house. Similarly, if there are children in the home, the lowest level bedrooms or other areas such as a playroom are preferred. Detectors should be placed ∼40 cm from an interior wall or ∼50 cm from an exterior wall. Among a total of 3215 measurements, 1775 tests were conducted in upper floors, 1294 in basements and 146 had no test location specified. While all tests were performed in basements in previous thoron studies, only ∼40 % of tests were located in basements in this study. However, by following the radon measurement protocol, this study provided a more realistic estimate of the radon and thoron exposure of the occupants. Nevertheless, the specified detector locations may be further away from potential thoron sources and resulted in relatively lower thoron concentrations due to the very short half-life of thoron gas.

One can see from Table 2, except in the NCR where radon characteristics agreed reasonably well among three studies, that fluctuations in radon characteristics were clearly observed in Halifax and Winnipeg from different studies/surveys. Such large fluctuations are not unusual for a sample size of ∼100 in a metropolitan area. Winnipeg is a city of 778 400. From 1983 to 1990, Health Canada conducted a case–control study for radon and lung cancer in Winnipeg^(19)^. There were 3669 tests conducted in basements and 4238 in bedrooms on upper floors. Averaged over ∼8000 measurements in basements and bedrooms, the average radon concentration in Winnipeg homes was estimated to be 159 ± 165 Bq m^−3^. The three subsequent studies had a sample size of only ∼100. Even with such small sample sizes, the estimated AM radon concentrations were well within the SD of the more accurate estimate of AM = 159 Bq m^−3^ determined from several thousand measurements. Repeated studies have demonstrated that radon concentrations in some Canadian homes are above the recommended action level of 200 Bq m^−3^. Taking the city of Winnipeg for example, >20 % of Winnipeg homes were >200 Bq m^−3^ 30 y ago, and it still is the case now.

## CONCLUSIONS

Strong evidence has demonstrated that domestic radon exposure causes lung cancer. ^222^Rn (radon) and ^220^Rn (thoron) are the most common isotopes of radon. In order to assess thoron contribution to indoor radon and thoron exposure, a survey of residential radon and thoron concentrations was initiated in 2012 in ∼4000 homes in the 33 metropolitan areas of Canada. The survey had a return rate of 79 %. It confirmed that indoor radon and thoron concentrations are not correlated and that thoron concentrations cannot be predicted from widely available radon information. The results showed that thoron contribution to the radiation dose varied geographically from 0.5 to 6.0 %. The study indicated that on average, thoron contributes ∼3 % of the radiation dose due to indoor radon and thoron exposure in Canada.

Even though the estimated average thoron concentration of 9 Bq m^−3^ (population weighted) in Canada is low, the estimated average radon concentration of 96 Bq m^−3^ (population weighted) is more than double the worldwide average indoor radon concentration (AM = 39 Bq m^−3^, population weighted)^(1)^. It is clear that continued efforts are needed to further reduce the exposure and effectively reduce the number of lung cancers caused by radon.
